# Computational Studies of Auto-Active van der Waals Interaction Molecules on Ultra-Thin Black-Phosphorus Film

**DOI:** 10.3390/molecules28020681

**Published:** 2023-01-09

**Authors:** Slimane Laref, Bin Wang, Xin Gao, Takashi Gojobori

**Affiliations:** 1 Computational Bioscience Research Center (CBRC), King Abdullah University of Science & Technology (KAUST), Thuwal 23955-6900, Saudi Arabia; 2 School of Chemical, Biological and Materials Engineering, Center for Interfacial Reaction Engineering (CIRE), University of Oklahoma, Norman, OK 73019, USA

**Keywords:** DFT, inhibitor, black-phosphorus, thermodynamic, molecular states, drug vehicles

## Abstract

Using the van der Waals density functional theory, we studied the binding peculiarities of favipiravir (FP) and ebselen (EB) molecules on a monolayer of black phosphorene (BP). We systematically examined the interaction characteristics and thermodynamic properties in a vacuum and a continuum, solvent interface for active drug therapy. These results illustrate that the hybrid molecules are enabled functionalized two-dimensional (2D) complex systems with a vigorous thermostability. We demonstrate in this study that these molecules remain flat on the monolayer BP system and phosphorus atoms are intact. It is inferred that the hybrid FP+EB molecules show larger adsorption energy due to the van der Waals forces and planar electrostatic interactions. The changes in Gibbs free energy at different surface charge fluctuations and temperatures imply that the FP and EB are allowed to adsorb from the gas phase onto the 2D film at high temperatures. Thereby, the results unveiled beneficial inhibitor molecules on two dimensional BP nanocarriers, potentially introducing a modern strategy to enhance the development of advanced materials, biotechnology, and nanomedicine.

## 1. Introduction

Aside from tumors, Ebola, Zika, and Influenza, the recent SARS-CoV-2 respiratory syndrome is an infectious disease that has killed millions of people worldwide since 2019. Two promising drug candidates for treatment are favipiravir (FP) and ebselen (EB) [1,2,3]. As an RNA polymerase inhibitor, 6–fluoro–3-hydroxypyrazine–2–carboxamide (C_5_H_4_FN_3_O_2_), commercially named favipiravir, is a modified pyrazine. Favipiravir has recently been developed to treat infections related to the Ebola virus. Ebselen (or 2–phenyl-1,2–benzoselenazol–3–one, C_13_H_9_NOSe) is one of synthetic organoselenium drug with antioxidant, cytoprotective activity, and is anti-inflammatory. During the clinical tests, these drugs have been reported to be safe [4,5]. Although, trials research on favipiravir and ebselen was conducted during the spread of SARS-CoV-1 and MERS–CoV infections, it was also claimed to be active against this virus in animal model systems [6]. These compounds have previously been investigated to treat multiple diseases such as influenza viruses [7,8,9,10], earshot loss [11,12] and bipolar syndromes [13,14,15,16]. Recently, in-vitro human cell studies reported that both favipiravir and ebselen could effectively stop the replication of SARS-CoV-2 at a very early stage [2,17,18].

Nowadays, numerous 2D Xenes materials have been comprehensively exploited widely in thermotherapy or as the delivery vehicle for anticancer medications [19,20,21]. Due to their exceptional and distinctive properties, two-dimensional (2D) materials have been exploited with great success [22,23,24,25]. Exhausted by the great success of graphene, a plethora of 2D nanomaterials have been elaborated and applied to a wide range of applications such as biomedical, catalysis, optoelectronic, and biotechnology [26,27,28,29,30,31,32]. However, transition metal dichalcogenides (TMDs), nitrides and carbonitrides (MXenes), hexagonal boron nitride (h–BN), and their derivatives were realized with few layers [33], which exhibit weak van der Waals interlayer bonding and strong in-plane covalent binding [34,35,36]. With nearly all atoms exposed to the ultrathin layer after exfoliation, the surface areas of these 2D materials are significantly increased, which improves their chemical reactivities and affects the 2D wave function from quantum confinement effects.

As well as the spotlight on their distinct electrical, phononic, photonic, and magnetic properties that set them apart from their bulk counterparts, ultrathin 2D nanocrystallines have a significant advantage in the aforementioned applications. In order to consider the biosafety and biocompatibility of Xenes 2D materials, including their biodegradability, cytotoxicity, and hazardous derivatives, deep research has been undertaken on their biological behavior [37]. The broad spectra of potential uses and clinical implementation of the Xenes 2D systems in biomedical drug therapy still require proper control of biodegradability [38] and toxicity, as well as an explicit understanding of the biochemical procedures during the loading of the particles on 2D nanomaterials and their interfaces. The first aspect is particularly valuable because the understanding of the interactions of polycyclic molecules with the layered materials can characterize the process of raising biologically friendly materials for gene silencing, scattering complexes, hybrid pharmaceutic ingredients, and biomedicine sensing, that can be described by knowing how aromatic fragments interact with layered materials.

Evolutionary monoelemental 2D materials have recently attracted a lot of attention [39,40], especially in light of the increased influence of phosphorene (known also as black phosphorus (BP)) and borophene, two materials with unexpectedly remarkable performance in biotechnology applications [41]. As a result, global challenges have arisen in the fields of the bioenvironment, healthcare, and harvesting energy. Phosphorene, in particular, has been regarded as a promising biological theranostic agent for diagnosis and optical therapeutic strategies (photodynamic and photothermal therapy [42], highlighting their biomedical applications in biomedicine owing to their excellent optical and electronic properties. Physical characteristics, such as an extremely high surface area to volume ratio, allow phosphorene and theranostic molecules to interact exhaustively on the surface. Those results, however, show an unusually high loading capacity [43], in which the conventional nanoparticle–based drug delivery platforms cannot be achieved. Additionally, rapid responses to external stimuli could be achieved due to the ultrathin 2D sheet and loaded molecules that could be released quickly [44]. The biological and physicochemical properties of black phosphorus enable the attachment of a variety of biological markers for biosensing applications, such as DNA, cellular growth hormones, hydrogen peroxide, and more [45,46]. The range of analysis and sensitivity of phosphorene-based biosensors can be improved by their possible chemical functionalizations and tunable electrical properties.

In the present study, motivated by the latest developments in the 2D nanomaterials synthesis and their encouraging solicitation for anti-tumor medications [47,48,49], we have performed a comprehensive study for the possibility of implementing 2D materials as nanocarriers for drug delivery in auto-treating therapy [50,51]. Therefore, by utilizing quantum chemicals, based on first-principles density functional theory (DFT), we report the structural stability, electronic and thermodynamic properties of FP and EB functionalized within a typical phosphorene 2D monolayer. The chemical bonding of FP and EB with 2D black phosphorus materials is revealed by considering the vacuum and continuum solvent model. Their influence on the orbital, charge transfer and optical gap properties was considered. Thereby, by calculating the Gibbs free energy changes of each step, we demonstrated that the temperature could be used to tune the releasing ratio of the aromatic molecules in a phosphorene sheet. Our results provide new fundamental insights into exploring innovative 2D materials as new, auto-active, likely nanocarriers for drug therapy. These atomistic results expand a contemporary method for the parametrization of multiscale approaches, including a coarse-grained method, Monte Carlo simulations, and molecular dynamics simulations, aiming to uncover the interaction of drugs/a 2D monolayer with the cell membrane as a function of their morphology.

## 2. Outcomes and Discussion

### 2.1. Structural Properties

The adsorption of favipiravir (C_5_H_4_FN_3_O_2_) and ebselen (C_13_H_9_NOSe) molecules on black phosphorus (BP) is studied on the most energetically stable configurations. To locate the most stable binding energy, the surface of a grid of (1 × 1) 0.25 Å was considered. The interaction of the molecules at each grid site was examined throughout geometry optimization, and the configuration corresponding to the lowest adsorption energy geometry was selected. Usually, for the adsorption of drugs onto biomaterial surfaces, there are many favorable sites that are available for adsorption; in this study, we only designated the lowest on energy. The binding energy, δ*E_Bind_*, of drugs on the surface is calculated as follows:

δEBind=ETot−(EBP+EMol)

where *E_BP_* is the energy of the surface, *E_Mol_* is the isolated energy of the molecule, and *E_Tot_* is the energy of the surface and the adsorbed molecule. A negative δ*E_Bind_* signifies stable adsorption. For simplicity, we refer only to the numerical values of binding energy in the entire manuscript. It is noted that we restrict the calculations to characterizing the adsorption of a single molecule on the respective surfaces. Consequently, the reported binding energies are in units of eV per molecule. The comprehensive interaction details of these drugs with black phosphorus will be consecutively presented. Below, we will discuss the relaxed surface under study and then focus on the different molecular anchors and their characterizations with the surface.

**Figure 1:**
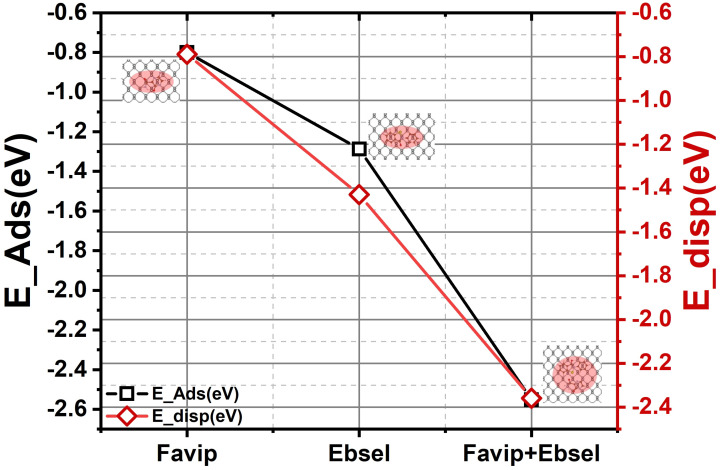
Adsorption and dispersion energy of favipiravir, ebselen, and favipiravir+ebselen hybrid molecules, respectively.

The phosphorene is the monolayer of black phosphorus, the main thermodynamically stable phase of phosphorus with an orthorhombic lattice, involving puckered layers of atoms [52,53]. To have the preferred stacking sites of the aromatic molecules and the 2D biomaterials, we explored the main favorite spatial configuration of the FP, EB, and hybrid FP+EB molecules on the nanocarrier. The molecular orientation results of the PB were studied through the analysis of the binding, formation, interaction, and covalent energies of the complex structures with different molecular orientations. All energetically favorable physisorption geometries of polycyclic molecules on pristine BP were identified as shown in Figure 1. Specifically, for FP, EB, and FP+EB molecules, they sat aligned on a zigzag row of the BP surface. However, the C_5_H_4_FN_3_O_2_ molecule was stabilized by the presence of carboxamide (–CONH2) moieties and the center of the pyrazine rings on top of the BP. In the case of the C_13_H_9_NOSe anchor, the molecule was stabilized by a di–benzene ring in the center of the BP surface, while carboxyl and selenazol moieties stayed away on the top of the hollow sites. Finally, FP+EB hybrid molecules showed the same planar preferred mode along with the adjacent BP monolayer. They were coupled throughout vdW interactions, as found in the previous case of FP and EB physisorbed alone on a pristine BP surface.

Energy minimized geometries of FP, EB, and FP+EB molecules with a BP sheet and key distances are illustrated in Table 1. It is apparent that C_5_H_4_FN_3_O_2_ energetically adopts a parallel orientation, whereas C_13_H_9_NOSe and hybrid molecules also have a planar orientation concerning the BP. This is related to the presence of carboxamide and selenium moieties strongly interacting with the P atom, respectively. The calculated vertical heights between FP, EB, and FP+EB molecules with BP are about 3.18, 3.28, and 3.24 Å, respectively. However, the binding energies for EF, EB, and FP+EB are −0.80, −1.29, and −2.55 eV, respectively. Interestingly, the simultaneous interaction between the two terminal functions and BP in the FP+EB molecules tend to be shortened over the BP nanocarrier to optimize the vdW forces (see Figure S1).

This can be understood by the decomposition of the interaction energy of pure GGA (covalently) and vdW dispersion energy terms that are mainly predominant in all configurations (*E*_vdw_^Hyb^ = −2.36 eV and *E*_Cov_^Hyb^ = −0.19 eV). The interaction energy of FP+EB (−2.15 eV) is much higher than FP (−0.82 eV) and EB (−1.31 eV) due to oxygen and selenium affinity in EP+EB with a lone pair of phosphorus atoms [52,53], and the phosphorene sheet is pulled out towards –CONH2 and –CONSe moieties in FP, EB, and hybrid molecules. Alternatively, by analyzing the energy decomposition (see Table 1), the binding energy is split into destabilized (positive) and minor deformation energies (FP, EB and BP sheet) and stabilized (negative) with predominant interaction energies between FP, EB, and FP+EB with BP (vdW interactions). The deformation energies of the BP sheet (calculated from the optimal deformed BP nanocarrier related to adsorption structures) are also weak for FP, EB, and hybrid molecules. In contrast, the deformation energy of hybrid FP+EB molecules (−0.44 eV) is more stabilized along with FP and EB molecules (see Figure S2).

Additionally, the optimized geometry of the three molecular forms on the BP sheet was carried out in aqueous medium states using the implicit solvation model [54], which was previously used for organic compounds [55]. The energy results of the FP, EB, and hybrid FP+EB in the water environment show weak stabilization of these molecules. This weak stabilization is due to the low atomic polarizability at contact with the solution phase. The calculated relative binding energies are −0.61, −1.26, and −2.06 eV for FP, EB, and FP+EB, respectively. The reduced relative binding energy of these aromatic molecules in a polar aqueous complex environment leads to a significant reduction in energy differences.

### 2.2. Electronic Properties

**Figure 2:**
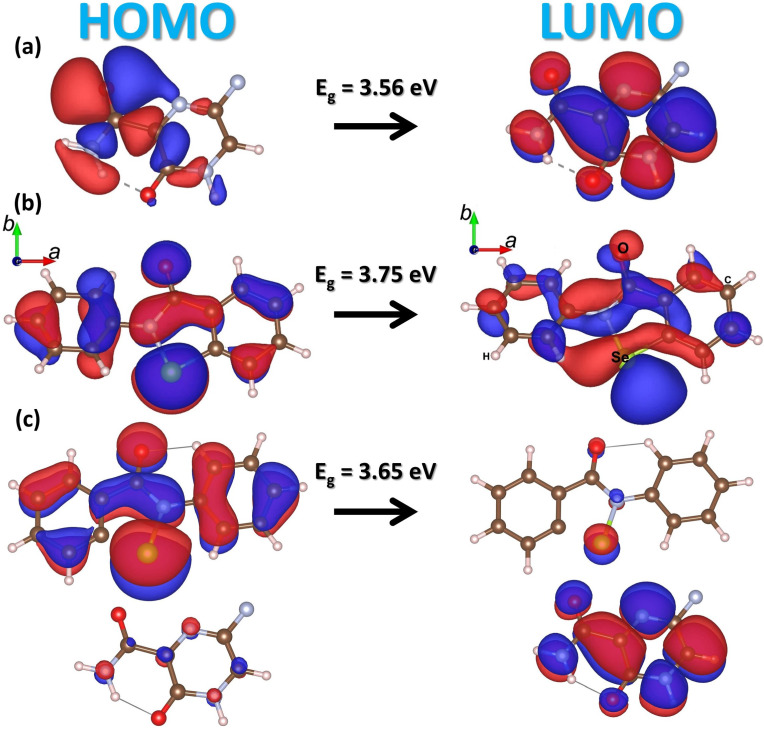
Three dimensional (3D)–view of the highest occupied (HOMO) and lowest unoccupied (LUMO) molecular orbitals and energy gap of (**a**) favipiravir, (**b**) ebselen, and (**c**) hybrid free molecules, respectively. The red and blue colors illustrate isosurface electron accumulation and depletion (ρ = ±9.5 × 10^−5^ē/Å^3^), respectively.

Figure 2 shows standalone molecular 3D plots of Kohn–Sham frontier orbitals and the calculated energy gap between the lowest unoccupied molecular orbital (LUMO) and the highest occupied molecular orbital (HOMO) by means of an HSE06 functional. It is noted that the HOMO orbitals are spread over all the molecules. In contrast, the LUMO states are localized on the pyrazine ring, carboxamide groups, and di–benzene, selenium–nitrene ring of C_5_H_4_FN_3_O_2_ and C_13_H_9_NOSe molecules, respectively. The calculated energy gap (E^HOMO–LUMO^) is 3.56 and 3.75 eV for FP and EB, respectively, and is in good agreement with the previous theoretical and experimental results [56,57]. Thus, the corresponding 3D and the optical energy of HOMO–LUMO states for FP, EB, and the hybrid FP+EB molecules are given in Figure 2.

The frontier molecular orbitals of antiviral molecules no longer have energy dispersion, and this suggests that there is not much hybridization between the molecules and the BP sheet. From these findings, it is clear that the HOMO–LUMO gap of the adsorbed FP, EB, and EP+EB on the BP sheet is located at the Fermi level, giving rise to a gap of 1.38, 1.37, and 1.35 eV, respectively. Likewise, these results reflect the interaction variations among the selected class of drugs. The HOMO–LUMO gap is in generally dominated by the biomaterials complex system. Further, the functional complexes also share similar HOMO–LUMO structures to those of the pristine phosphorene, except for the hybrid FP + EB on the BP nanocarrier. An exception is that, due to the strong interactions between the hybrid molecules and BP, a new state appears above the Fermi level of the BP; i.e., the lowest state is located by the LUMO of the FP molecule. Moreover, it is noted that, in the aqueous phase, HOMO–LUMO is increased, and the gap is about 1.45, 1.44, and 1.44 eV with respect to FP, EB, and FP+EB adsorbate states.

**Figure 3:**
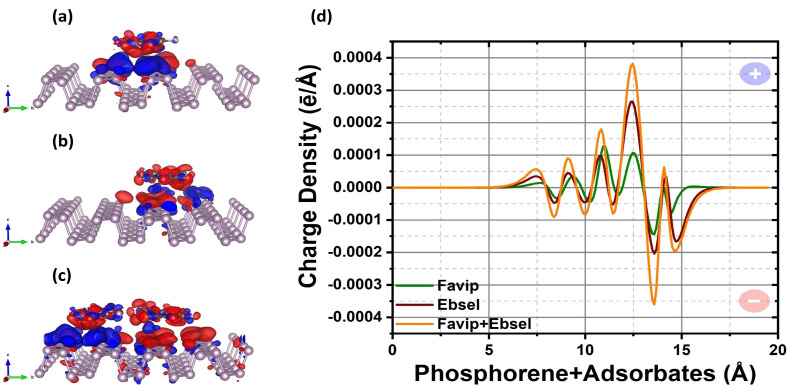
(**a**–**c**) Three dimensional (3D)–view of the electronic charge density difference δρ for favipiravir, ebselen, and hybrid molecules on phosphorene sheet (δρ = ±3.5 × 10^−4^ ē/Å^3^). The red and blue colors indicate isosurface electron accumulation and depletion, respectively. (**d**) Corresponding 1–D averaged electron–density difference (δρ(*z*)) along the adsorption direction for favipiravir (green line), ebselen (wine line), and hybrid (orange line) favipiravir+ebselen molecules on phosphorene sheet.

The charge redistribution plots reveal a small electron transfer between the FP, EB, FP+EB and BP surfaces as represented in Figure 3 due to the physisorption. The adsorption of FP and EB molecules on the phosphorene surface leads to electron transfer from the phosphorene towards the molecules (see Figure 3a–c). Additionally, each phosphorus atom is covalently bonded to three adjacent phosphorus atoms to form a puckered honeycomb structure in pristine BP. By forming three bonds with neighboring P atoms, each phosphorus atom also has a lone pair of electrons. For the FP/phosphorene, the differential charge density shows a spatial electronic fluctuations redistribution in the functional phosphorene when the FP is close to the surface. Once more, the interaction between the adsorbed fragment and the BP single layer creates electron transfer from the phosphorene monolayer to the physisorbed drugs, thus spacial electrons located on the anchor indicate that additional P atomic bonds are provided for electrons to lift through adsorbed molecules continuously. Likewise, the vdW interaction has predominantly induced the associated degree of the P–P bond in the long conjugated π states of phosphorene film, which is surrounded by the atomic layer of phosphorus and the adsorbed molecules.

**Figure 4:**
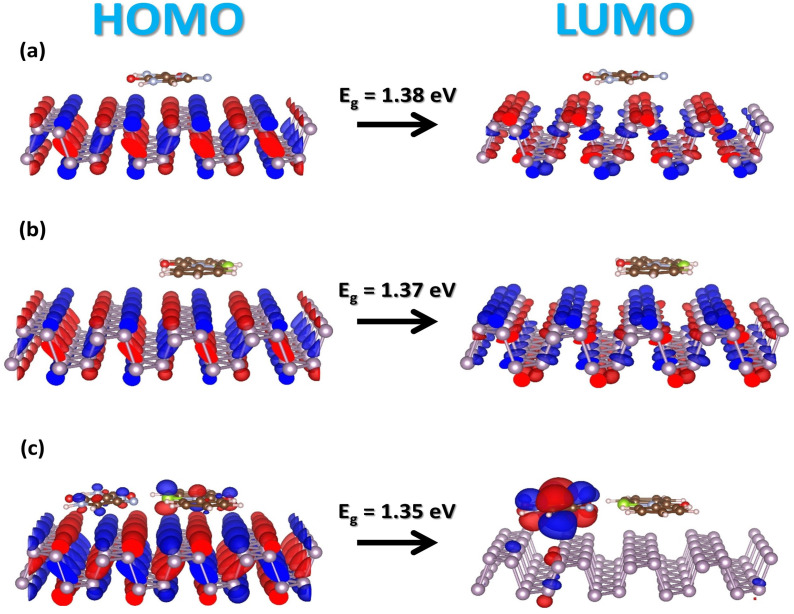
Three dimensional (3D)–view of the highest occupied (HOMO) and lowest unoccupied (LUMO) orbitals, and energy gap of (**a**) favipiravir, (**b**) ebselen, and (**c**) hybrid molecules on black phosphorus. The red and blue colors represent isosurface electron accumulation and depletion (ρ = ±3.5 × 10^−4^ ē/Å^3^), respectively.

**Figure 5:**
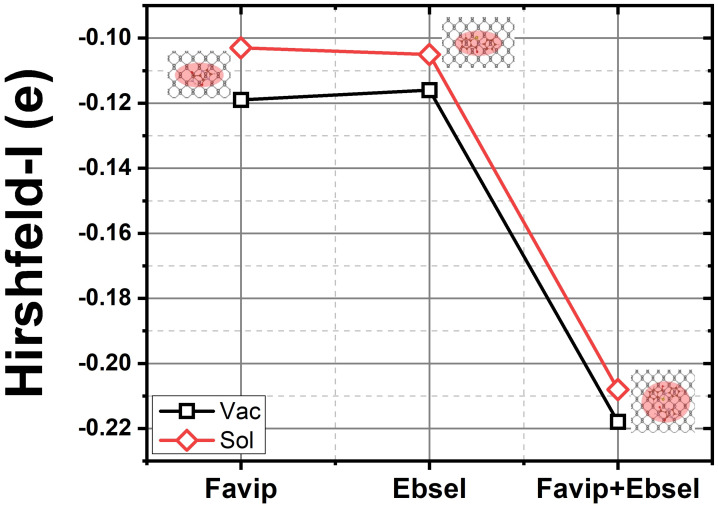
The calculated Hirshfeld–I charge transfer from BP sheet to respective favipiravir, ebselen, and favipiravir+ebselen hybrid molecules in a vacuum (black) and with solvent (red).

Electron accumulation (red) and depletion (blue) are observed near the region between BP and the FP molecules. The electrophilic groups of FP attract the electrons from the 2D materials and polarize the complex underneath the adsorption site, causing the intramolecular charge relocation. Specifically, in Figure 4, the lone pair of the 3*p* orbitals of the P atom within the phosphorene is highly perturbed by the transferred charge. For those phosphorus atoms located near the pyrazine and carboxamide groups of the FP molecules, the valence electron population decreases the most as the oxygen and nitrogen atoms have the strongest electronegativity along with all atoms in the system. As shown in Figure 5, from the Hirshfeld–I charge analysis, FP gains charges of −0.12 ē with respect to a standalone molecule (see Figure 2). The unperturbed orbital characteristics of BP (nucleophilic phosphorus atom) upon adsorption of FP and EB antiviral molecules may drive fragments to be the desired functional groups of the phosphorene monolayer.

There are different molecular bonding characteristics of phosphorene; the larger adsorption energy of the aromatic molecules on the BP may vary due to its strong in-plane Coulomb interactions. To figure out the mechanisms for the enhanced physisorption strength of the FP and EB compared to FP+EB hybrid cases on phosphorene, we considered the average planar electronic density difference as illustrated in Figure 3d for FP/BP, EB/BP, and FP+EB/BP, respectively. The calculated dipole moment of FP and EB on phosphorene in a vacuum (with solvent) is, respectively, 1.40(1.39) and 4.36(4.41) a.u, which is related to the inducing polarizability from the charge transfer of the EB molecules. The fluctuation of planar electronic density between the phosphorus atoms and favipiravir is larger, and ebselen cancels part of the polarization, creating smaller polarization values in the FP/phosphorene interface. The EB has larger polarizability on BP, suggesting a similar influence of physisorbed inhibitor molecules on the 2D monolayer black phosphorous.

**Figure 6:**
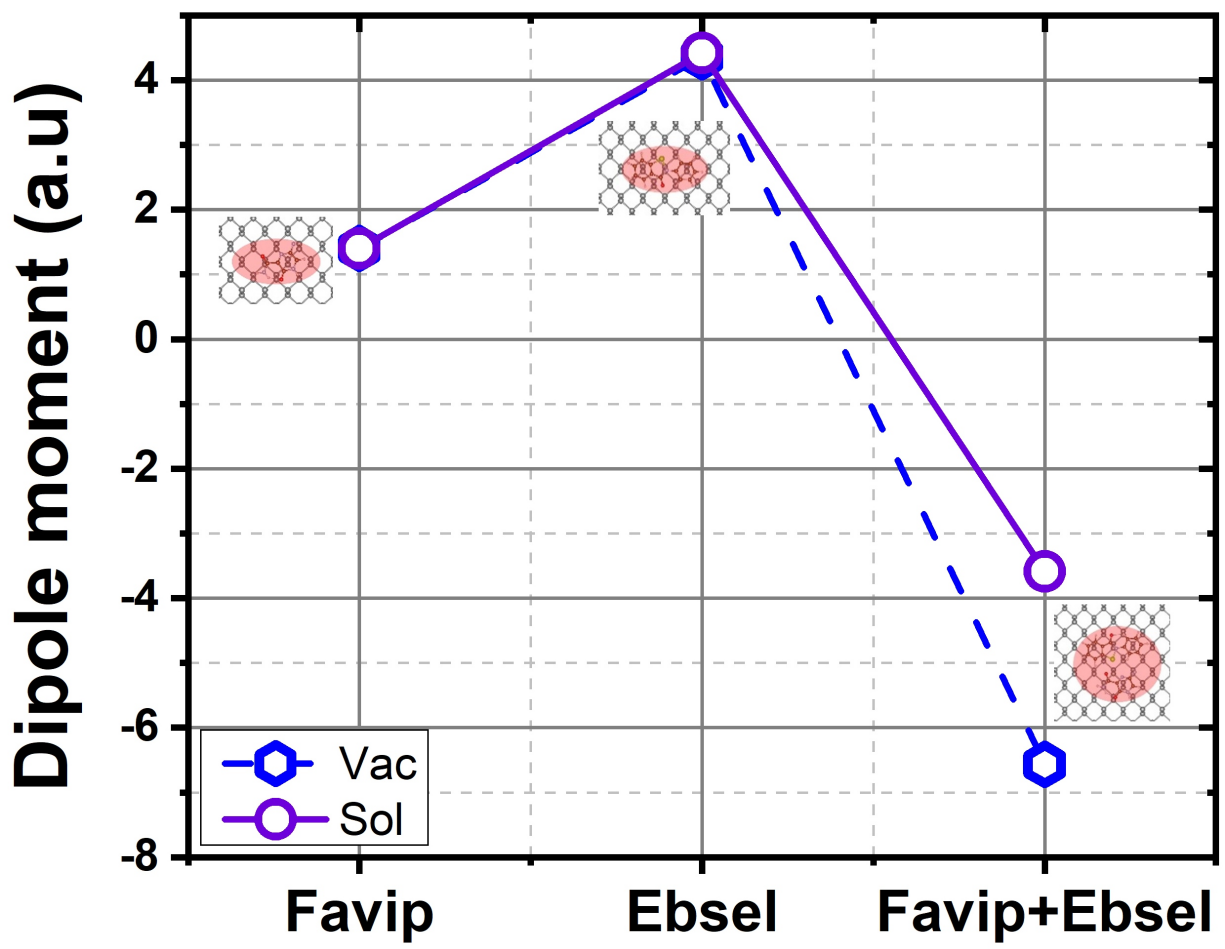
The calculated dipole moment of BP sheet with favipiravir, ebselen, and favipiravir+ebselen hybrid molecules in a vacuum (blue) and with solvent (purple), respectively.

Interestingly, for the case of hybrid FP+EB, the change of the orbitals (see Figure 4c) is induced by a strong polarizability in the combined molecules (dipole moment is −6.56 (vacuum) and −3.60 (solvent) a.u; for more details, see Figure 6). Consequently, the enhanced physisorption ability can be illuminated by the interplay between the nondirectional charge fluctuations and the induced atomic polarizability of the drug molecules and the 2D material interface. Therefore, both the vdW forces and in-plane electrostatic interactions affect the characteristics of the chemical strength.

**Figure 7:**
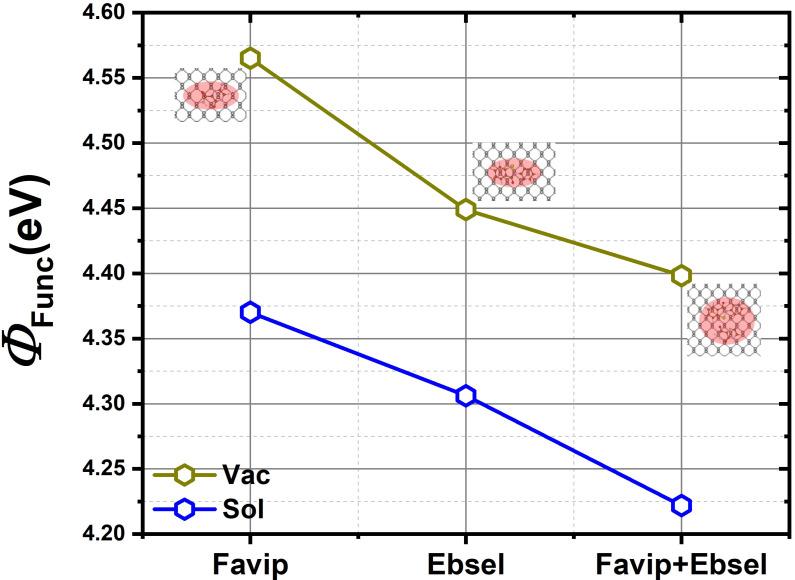
The calculated work function Φ in vacuum and solvent phases of BP sheet with favipiravir, ebselen, and favipiravir+ebselen hybrid configurations in a vacuum (olive) and with solvent (blue), respectively.

In addition, we have calculated the change in the electrostatic potential that is produced by antiviral molecules and the black phosphorus sheet. The electrostatic potential profiles represented in Figure 6 show a downward shift from FP to EB molecules, demonstrating an electron withdrawing in the EB molecule. Nonetheless, the –CONSe moieties in EB results in reduced work function compared to the –CONH2 groups of FP on BP. In Figure 7, the calculated work function of FP/BP is 4.56 eV is very close to the computed value of the pristine BP (4.59 eV). The work function of EB/BP is reduced by 4.45 eV. The adsorption of FP and EB molecules on the phosphorene layer lowers the work function to 4.40 eV. When FP and EB are added together onto the 2D material, the drop in the work function is directly proportional to the interface dipole moment per unit area (Figure 6). Further, Figure 4a–c illustrates the Kohn–Sham frontier orbitals; from the 3–D view molecular orbitals in Figure 4c, it is perceived that there is a significant depletion of electrons adjacent to the aromatic side of the molecules and a large accumulation of electrons close to FP at the BP interface. This large interface dipole arises from the adsorbed molecule pulling electrons out of the BP sheet, reducing the work function by 0.19 eV. On the other hand, the slight buckling of the BP sheet gives rise to another dipole moment that potentially cancels the interface dipole moment mentioned above (see Figure S4). This gives rise to a smaller work function change in the BP systems upon FP and EB physisorption, which is fairly consistent with previous findings related to other aromatic molecules on BP [52,53,58]. Moreover, the aqueous solvent phase further reduces the work function to a value of 4.34, 4.31, and 4.22 eV for FP, EB, and FP+EB molecules on the BP sheet, respectively.

## 3. Thermodynamic Properties

**Figure 8:**
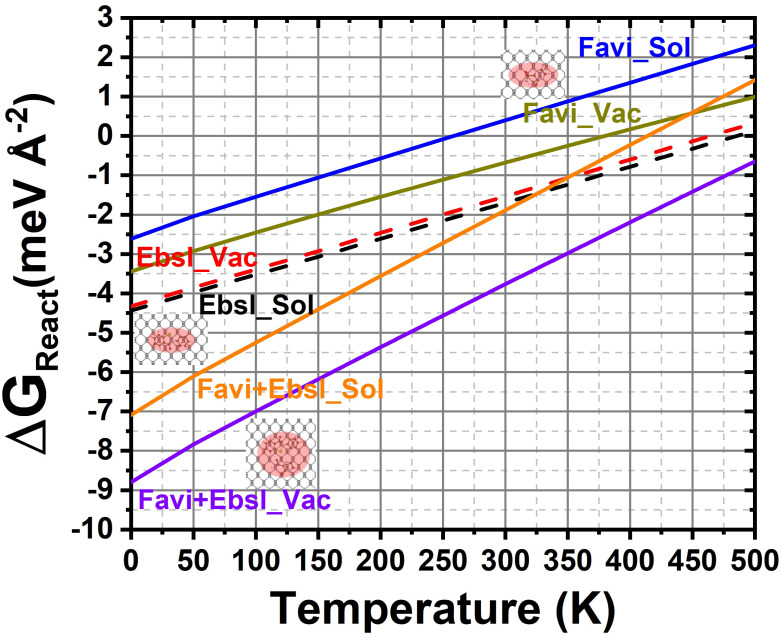
The free Gibbs energy process as a function of temperature in the vacuum and solvent phases of BP sheet with respect to favipiravir, ebselen, and favipiravir+ebselen hybrid configurations.

The structural, energetic, and electronic properties of FP, EB and hybrid FP+EB molecules were described above. The next steps in the study involved the key role of temperature-dependent adsorption behavior. Ideally, the aromatic molecules are released from the surface due to the external stimulus such as rising temperatures; this process was simulated by calculating the Gibbs free energy of the complex with and without the drug. The Gibbs free energy changes for all intermediate configurations during the loading and releasing process are calculated as follows:

ΔG=ΔEDFT+ΔEZPE+TΔS

where Δ*E_DFT_*, Δ*E_ZPE_*, and TΔ*S* represent the difference of DFT-calculated binding energy, zero-point energy (ZPE), and entropy contribution (*S*), respectively. Accordingly, as illustrated in Figure 8, the temperature indeed affects the reaction energy of the drugs on the 2D biomaterial (vacuum/solvent–biomaterial phase). It is well noted that the vibrational correction shifts the adsorption to more positive or fewer negative values at a higher temperature. The zero-point energy at lower temperature values (0–100 K) has a minor effect on the energy (between 1 and 2 meV/Å^2^ for FP, EB, and FP+EB, respectively). On the other hand, increasing the temperature from 100 to 430 K shifts the energy up to 3.5 to 4 meV/Å^2^ for FP, EB, and FP+EB on the BP–vacuum interface with the inclusion of vibrational free energy. The same trend was illustrated when including implicit solvent in this complex system. Remarkably, for hybrid FP+EB on a BP sheet (vacuum phase), the adsorption remains stable up to 450 K. The energy deviations indicate that, during the adsorption process, the thermotherapy is enthusiastic, and the releasing rate of the inhibitor drugs will be greatly enhanced due to the large drop in the binding energy at higher temperatures.

In general, those molecules will have a larger releasing rate at a higher temperature; this could be due to the superior thermal variation at a higher temperature. In this context, related to the ex-vivo evaluation of the tumors’ process, the BP-loaded molecules can be used as an anti-defected membrane treatment that has a pertinent action in the thermal therapeutic. The temperature could increase up to 450 K within a few minutes. The excellent therapeutic efficiency of these drugs in the BP-based complex is closely related to the high ability to target drug release and the hyperthermia of the infected cell. Overall, these results indicate that the hybrid FP+EB therapy of the 2D biomaterial-based delivery systems can be effectively activated. These studies will contribute to optimizing virus growth and storage conditions, facilitating the molecular characterization of different pathogens. The benefit of the antiviral molecules with a phosphorene sheet as a new auto-treating delivery scheme is that it could continuously control the molecular adsorption and desorption free energy of the polycyclic molecules by applying a temperature, inducing external stimuli for changing its temperature.

## 4. Methodology

All DFT calculations were carried out using the Vienna *Ab initio* Simulation Package (VASP 5.4) [59,60]. All electrons projecting augmented wave (PAW) formalism were used to model the electron–ion interactions [61]. The exchange–correlation functional contribution to the total energy was modeled using the generalized gradient approximation (GGA). In addition to the modified Perdew–Burke–Ernzerhof (PBE) exchange–correlation functional [62] for treating the van der Waals (vdW) interaction corrections through the D3–BJ approach was incorporated in all calculations, as established by Grimme et al. [63,64,65]. We used a (5 × 4) supercell model of a BP monolayer containing 80 phosphorus atoms for the nanocarrier to adsorb C_5_H_4_FN_3_O_2_, and a C_13_H_9_NOSe anchor. A vacuum region of 20 Å was introduced perpendicular to the BP film to prevent unphysical periodic effects. Given the large size of the supercell, a numerical integration was employed over the Brillouin zone using a 3 × 3 × 1 Γ–centered k–point grid [66]. During the self-consistent field (SCF), frontier orbitals, charge density transfers, and optical gap calculations, a denser 5 × 5 × 1 mesh was applied. The pseudo-wave functions were expanded in a plane-wave basis set with an energy cut-off of 450 eV. The accuracy was ensured by adopting energy-convergence criteria of 1 × 10^−5^ eV. Therefore, structural relaxation was performed using the conjugate gradient technique until the force on each atom was below ±0.001 eV/Å. This strategy was also employed previously with success to characterize fullerene adsorption on single-layer graphene [38]. Further, this study exhibits the quantification of antiviral molecules on a phosphorene monolayer that can deliver comprehensive insight via quantified interaction energy.

## 5. Conclusions

We performed a comprehensive density functional van der Waals approach to investigate the interaction peculiarities of antiviral molecules on a single layer of a phosphorene sheet. We carried out *ab initio* calculations in a vacuum and with a continuum solvent level to assess the binding characteristics and thermodynamic properties for auto-active inhibitor drug delivery. We note that favipiravir together with ebselen molecules would empower functionalization of the 2D phosphorene and permit thermostability at room temperature. Furthermore, both molecules displayed a strong van der Waals interaction when combined with the black phosphorous sheet. It is important to note that the hybrid FP+EB adsorption state exhibits a more pronounced adsorption energy due to the Coulomb interactions, and gain on electron transfers. The results reported in this work indicate that the quasi-planar molecules and the intrinsic nucleophilic property of phosphorus atoms are intact. However, the change in the Gibbs energy at different temperatures demonstrates that these molecules facilitate the delivery of the antiviral drug from a single layer of phosphorene surface even in high temperature conditions. Our calculations are a fundamental insight toward rigorous van der Waals interactions in complex systems and will contribute, after experimental validation, to the development of new drugs based on quantum simulation and high throughput tools that are independent of any external input data. Finally, this finding suggests that these inhibitor molecules and 2D biomaterials are a new effective way to conceivably apply thermotherapy and obtain precise self-treating drug vehicle delivery.

## Figures and Tables

**Table 1:** The drug–BP adsorption distance d–d (in Å), van der Waals energy, binding energy, interaction energy, and covalently energy (in eV), corresponding to the different drugs identified in this work as evaluated by the PBE–D3(BJ) method.

	d–d	E_vdW_	E_Bin_	E_Int_	E_Cov_
FP	3.18	−0.79	−0.80	−0.82	−0.01
EB	3.28	−1.43	−1.29	−1.31	0.14
FP + EB	3.24	−2.36	−2.55	−2.15	−0.19

## Data Availability

No data available.

## Supplementary Materials

The following supporting information can be downloaded at: https://www.mdpi.com/article/10.3390/molecules28020681/s1, Figure S1: The drug-BP van der Waals energy, binding energy, interaction energy and covalently energy, of favipiravir, ebselen and favipiravir+ebselen hybrid drugs respectively; Figure S2: The drug-BP slab deformation energy, drug deformation energy, and covalently deformation energy, of favipiravir, ebselen and favipiravir+ebselen hybrid drugs respectively; Figure S3: The free atomic polarizability, with respect of drug-BP favipiravir, ebselen and favipiravir+ebselen hybrid drugs; Figure S4: The calculated 1-D electrostatic potential profile of favipiravir (black line), ebselen (blue line) and favipiravir+ebselen (red line) hybrid drugs with respective BP sheet.

## Sample Availability

Samples of the compounds are not available from the authors.
