# Supplementary material for: Computational Studies of Auto-Active van der Waals Interaction Molecules on Ultra-Thin Black-Phosphorus Film
Source: Molecules. 2023 Jan 9;28(2):681. doi: 10.3390/molecules28020681 (PMC9864666; doi:10.3390/molecules28020681)
Supplement: Supplementary file 1 [file molecules-28-00681-s001.zip › molecules-2138302-supplementary.pdf]

Supplementary Materials

Computational Studies of Auto–Active van der Waals interaction Molecules on Ultra–thin Black–Phosphorus Film

Slimane Laref [1,*], Bin Wang [2,†], Xin Gao [1,†] and Takashi Gojobori [1,†]

[1] Computational Bioscience Research Center (CBRC), King Abdullah University of Science & Technology (KAUST), Thuwal 23955-6900, Saudi Arabia; xin.gao@kaust.edu.sa (X.G.); takashi.gojobori@kaust.edu.sa (T.G.)
[2] School of Chemical, Biological and Materials Engineering, Center for Interfacial Reaction Engineering (CIRE), University of Oklahoma, Norman, OK 73019, USA; wang_cbme@ou.edu

* Correspondence: slimane.laref@kaust.edu.sa

† These authors contributed equally to this work.

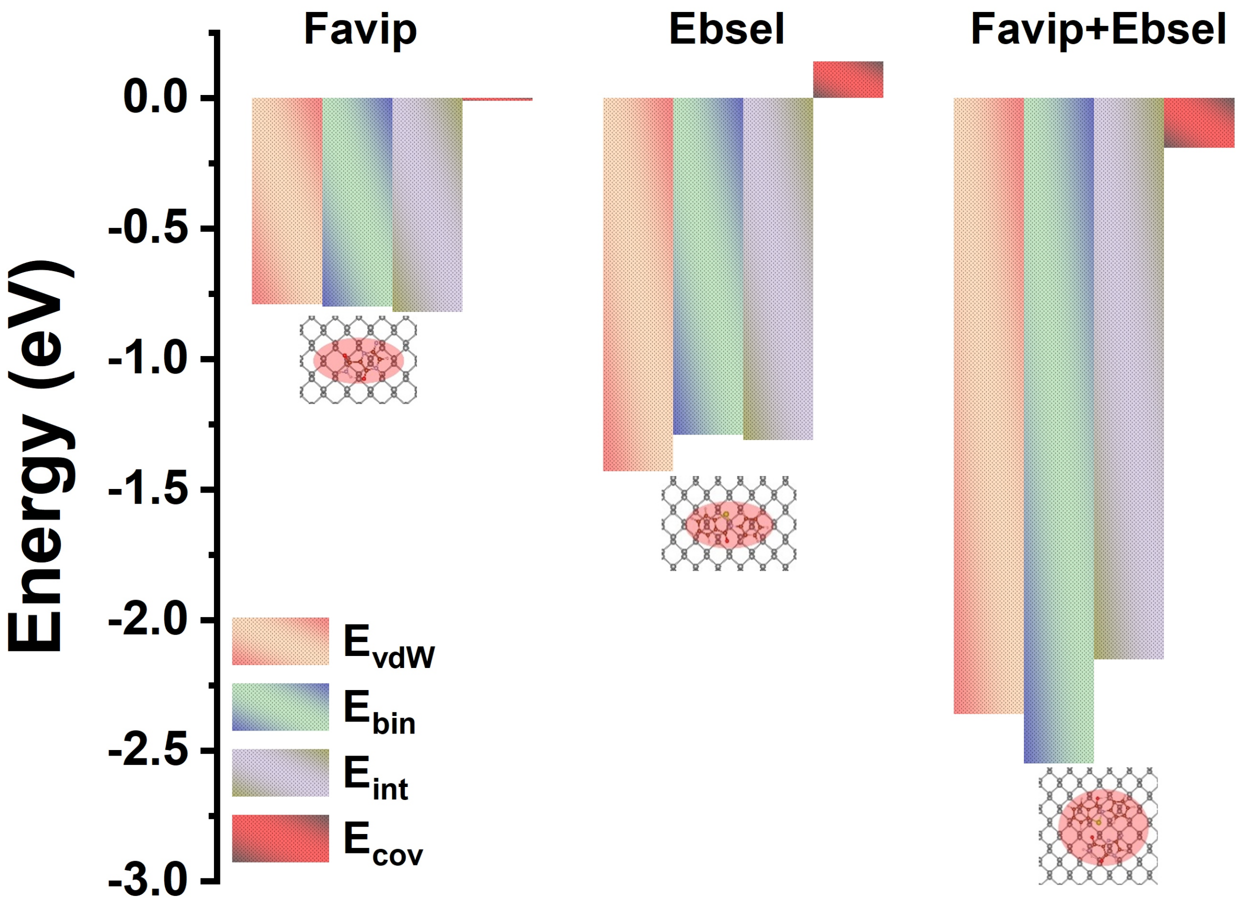

Figure S1. The drug-BP van der Waals energy, binding energy, interaction energy and covalently energy, of favipiravir, ebselen and favipiravir+ebselen hybrid drugs respectively.

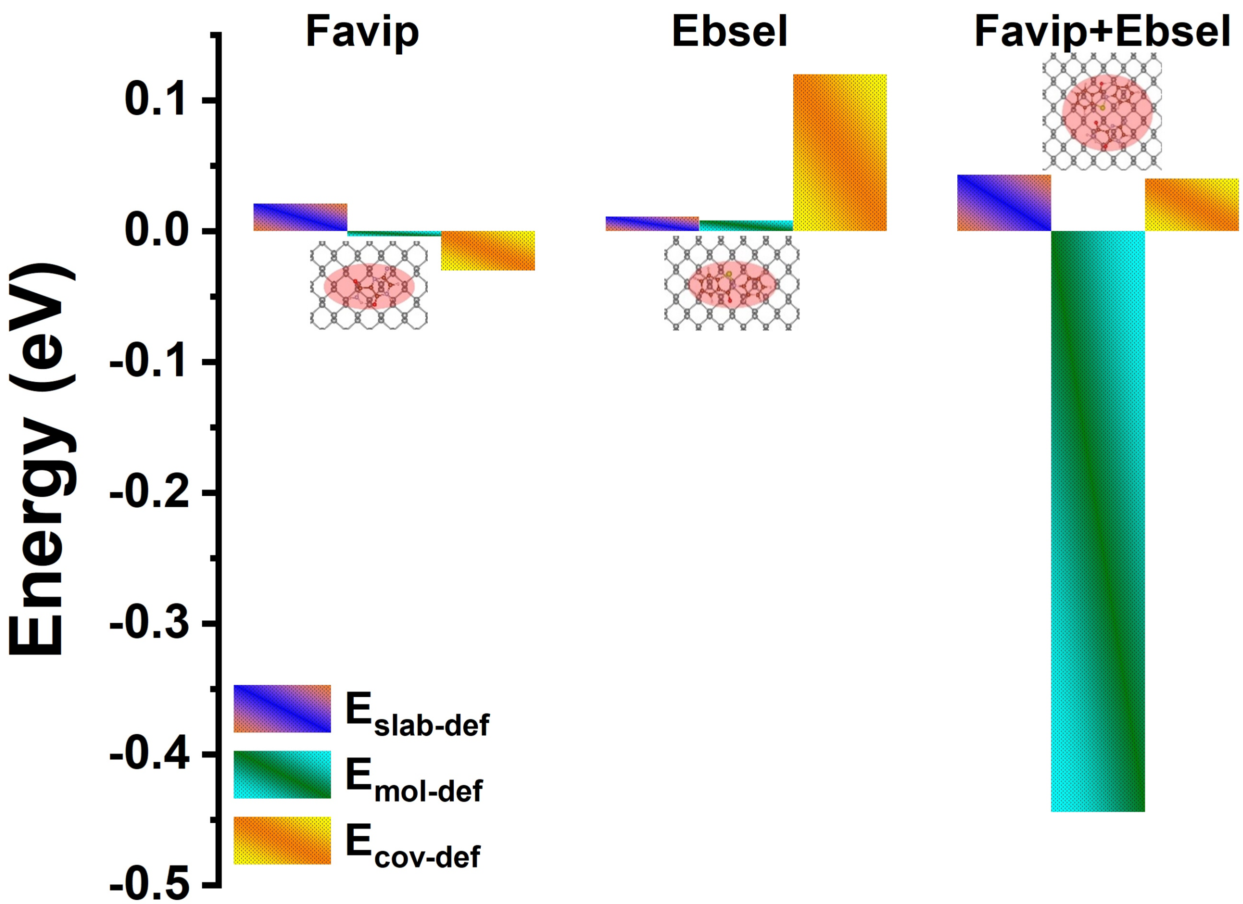

Figure S2. The drug-BP slab deformation energy, drug deformation energy, and covalently deformation energy, of favipiravir, ebselen and favipiravir+ebselen hybrid drugs respectively.

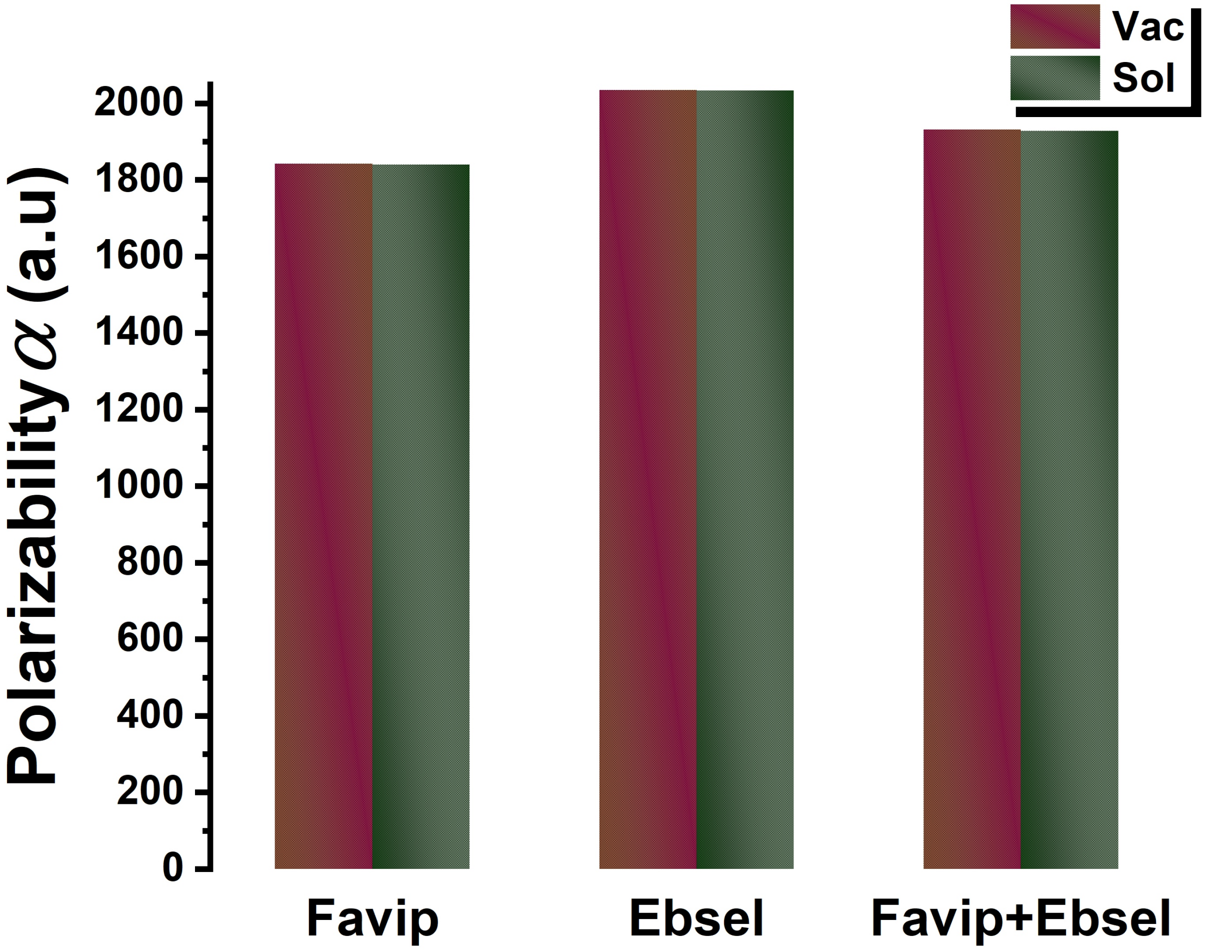

Figure S3. The free atomic polarizability , with respect of drug-BP favipiravir, ebselen and favipiravir+ebselen hybrid drugs.

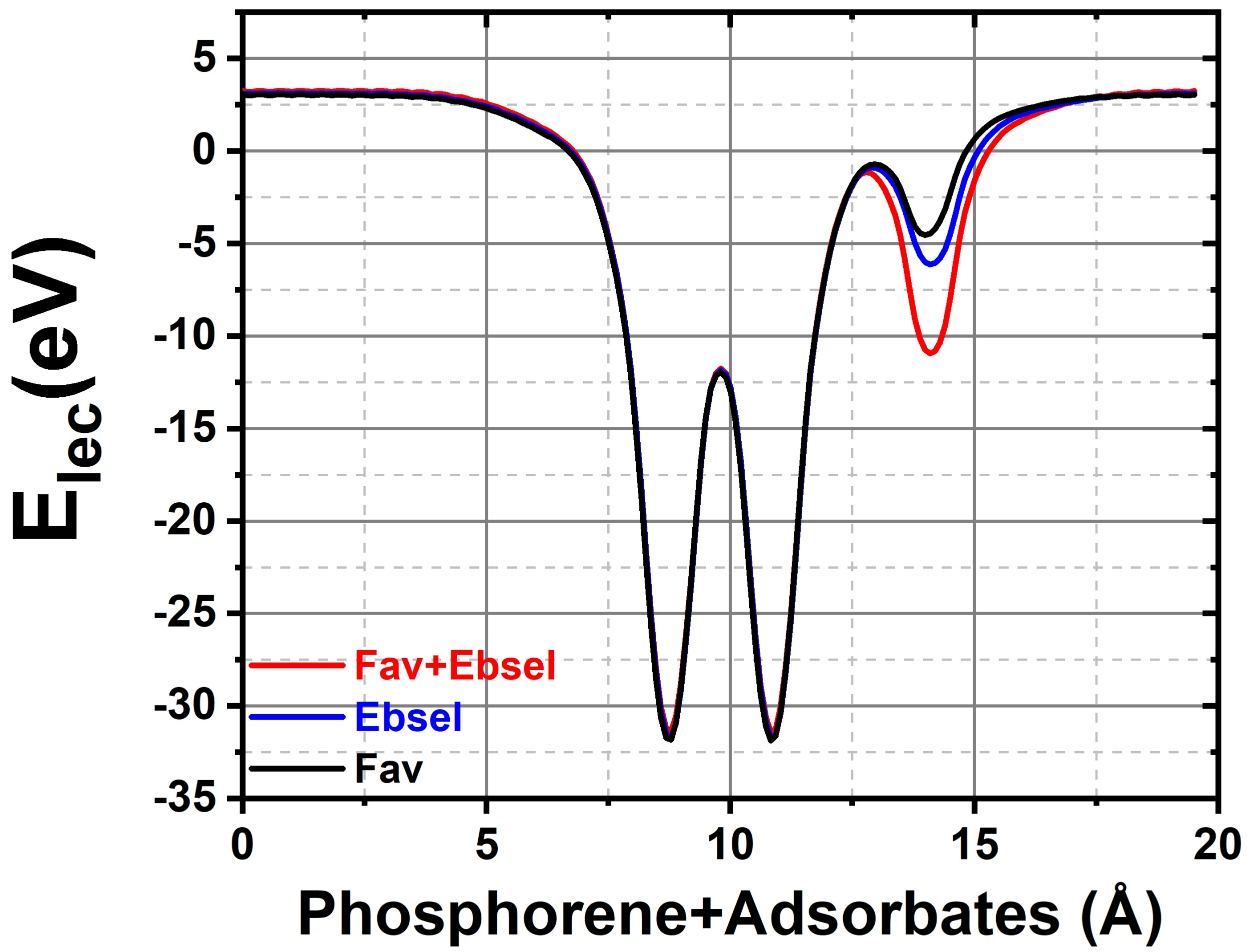

Figure S4. The calculated 1-D electrostatic potential profile of favipiravir (black line), ebselen (blue line) and favipiravir+ebselen (red line) hybrid drugs with respective BP sheet.
